# Routine Pap smear screening in Pakistan: A current perspective

**DOI:** 10.1016/j.amsu.2022.104498

**Published:** 2022-09-21

**Authors:** Simran Bhimani, Aashish, Govinda Khatri, Mohammad Mehedi Hasan

**Affiliations:** a Dow University of Health Sciences, Karachi, Pakistan; b Department of Biochemistry and Molecular Biology, Faculty of Life Science, Mawlana, Bhashani Science and Technology University, Tangail, Bangladesh

Cervical cancer is the second most common cancer in women under the age of 50 and the most common cause of mortality among women worldwide, especially in developing countries [1,2]. Pakistan has a population of around 60.6 million women belonging to the age group 15 and above, who are at the highest risk of developing this cancer [3]. Furthermore, current estimates indicate that every year over 5008 women are diagnosed with cervical cancer and approximately 3197 women die from the carcinoma, primarily due to the lack of knowledge, routine screening, prevention, and vaccination. Therefore, this led to more than half of the patients being diagnosed at a very advanced and unpreventable stage of malignancy [3].

Various etiologies of cancer can be categorized into unprotected and early sex, poor socioeconomic status, early reproductive cycles and multiparity, HPV infection, smoking, and low education level. The most common association amongst these is HPV, which is a virus spread by sexual transmission. The prevalence of this virus is extremely high in Pakistani women, especially because almost 88% with precancerous lesions of the cervix are HPV positive [2].

The long preinvasive stage which makes it a preventable disease (lasting for 20 years) leads to early detection and eventually an appropriate treatment if adequate screening techniques are implemented. The primary screening test, that is, pap smear test, either liquid-based or conventional smear methods, are considered for the detection of precancerous cervical intraepithelial neoplasia (CIN) and the early stage of invasive cancer. Pap smear identifies the epithelial cell abnormality which is further proceeded by colposcopy and biopsy. As aforementioned, colposcopy is a binocular microscope that visualizes the cervix and further abnormalities caught by this procedure lead to biopsy [4]. However, due to a lack of awareness and hurdles in cytology-based screening programs in Pakistan, this makes cancer unpreventable. Whereas, the integration of pap smears in developed countries has made cervical cancer from one of the most common gynecologic cancer to one of the least. Moving on, the procedure of a pap smear is simple economical, yet painless; first a speculum is placed inside a woman's vagina and identifies the cervix. Furthermore, the liquid-based method is in which the cells are collected from the transformation zone by the usage of a brush and placed into the liquid preservative. Additionally, in the conventional method, the collection of cells is done via brush and spatula and transferred to the slide with a preservative. Moreover, the liquid-based technique is above the conventional method for several reasons; by a single collection we can detect HPV, gonorrhea, and chlamydia, easier to interpret, results are almost accurate, and blood and debris are filtered [4]. To provide relief from the discomfort, the use of either water or a small quantity of water-based lubricant is used. Additionally, a standard system known as the Bethesda system is used for analyzing pap smears [4].

However, HPV infection is detected via polymerase chain reaction (PCR) [2]. Moreover, HPV vaccination is available for only certain subtypes of HPV which includes 6,11,16,18 but not a lot of other high-risk HPV infections. Along with that the vaccination only covers squamous cell carcinoma and not adenocarcinoma. Measures can be taken to prevent the disease in various ways that are being applied in developed countries. There is an urgent need in Pakistan due to ignorance about cervical cancer, HPV vaccination, prevention, and cultural myths and beliefs. The Ministry of Health of Pakistan can devise a standard cervical screening program and awareness campaigns (Pap smear testing) starting from the age group of 21 and should be integrated into routine primary health care services [1]. Along with this, according to the screening guidelines, implemented by the American Cancer Society (ACS) and American College of Obstetricians and Gynecologists Committee (ACOG), the initiation of screening should begin at 21 years of age, as it is considered a reproductive age and women can be infected by HPV after sexual intercourse. Moreover, women from the age of 21 should receive HPV vaccination to prevent the predisposing factor for cancer. Screening and vaccination are highly recommended in Pakistan, where women are married at an early age.

Additionally, awareness should be provided by encouraging women and their families to regular screening and educating them regarding the consequences of the diseases by using a locally understood language [5]. The doctors should be trained, and the facilities should be provided for outpatient treatment, appropriate follow-up care, easy access to healthcare for all categories of the population, and good technical and laboratory expertise [4,5]. Moreover, women from the age of 21 should receive HPV vaccination to prevent the predisposing factor for cancer. While there is a lack of knowledge, women in Pakistan often overlook their health. Therefore, raising awareness will result in a high yield of success of screening programs in Pakistan.

## Ethics statement

N/A.

## Source of funding

N/A.

## Author contributions

All authors meet the inclusion criteria, and all authors read and approved the final version of the manuscript.

## Trail registry number

N/A.

## Guarantor

Mohammad Mehedi Hasan, Department of Biochemistry and Molecular Biology, Faculty of Life Science, Mawlana Bhashani, Science and Technology University, Tangail, 1902, Bangladesh. Email: mehedi.bmb.mbstu@gmail.com.

## Declaration of competing interest

N/A.
